# Retention of a Double-J Stent Upper Coil in the Renal Parenchyma Following Percutaneous Nephrolithotomy: A Case Report

**DOI:** 10.7759/cureus.84981

**Published:** 2025-05-28

**Authors:** Ahmed Abdelrasheed, Adrian Sale, Muhammad Iqbal

**Affiliations:** 1 Urology, Royal Glamorgan Hospital, Pontyclun, GBR

**Keywords:** complications of jj stent, endoscopic approach, malecot catheter, pnl, renal calculi (kidney stones)

## Abstract

Percutaneous nephrolithotomy (PNL) is a standard treatment for large renal stones, and the use of a double-J (JJ) stent is common for postoperative drainage. Here, we report a rare complication involving retention of the upper coil of a JJ stent within the renal parenchyma following PNL. A 47-year-old man underwent left-sided PNL with placement of an antegrade JJ stent and Malecot catheter. Although initial imaging confirmed correct stent positioning, subsequent attempts at stent removal failed. Ureteroscopy revealed that the upper coil was embedded in the renal parenchyma, likely at the site of the prior puncture. Laser dissection was required to release the stent, which was successfully removed and replaced. This case highlights the potential risk of stent misplacement when used with a Malecot catheter and underscores the importance of fluoroscopic guidance during catheter removal and consideration of stent repositioning to prevent similar complications.

## Introduction

The incidence and prevalence of kidney stones are increasing globally across sexes, races, and age groups [1], affecting approximately 8% to 12% of the world population [2-6]. Percutaneous nephrolithotomy (PNL) is the first-line treatment for large, multiple, or inferior calyceal renal stones, according to the European Association of Urology guidelines [7]. Double-J (JJ) stents are fundamental and widely used in urological procedures to promote edema resolution and maintain ureteral patency [8]. The indications for JJ stent placement have expanded with advancements in endourological procedures. Absolute indications include obstructive pyelonephritis and severe, unrelenting renal colic [9]. Additional safety indications include ureteral edema, perforation, or steinstrasse following endoscopic procedures, history of renal failure, and solitary or transplanted kidney [10]. A well-documented complication of PNL is perforation of the renal collecting system, which may necessitate cessation of the procedure and placement of a perirenal drain, JJ stent, nephrostomy tube, or a combination of these interventions [11]. This report describes a rare complication involving retention of the upper coil of a JJ stent within the renal parenchyma after removal of the Malecot catheter, which is a self-retaining tube used in the drainage of different body fluids, e.g., urine, bile, and pus.

## Case presentation

**Figure 1:**
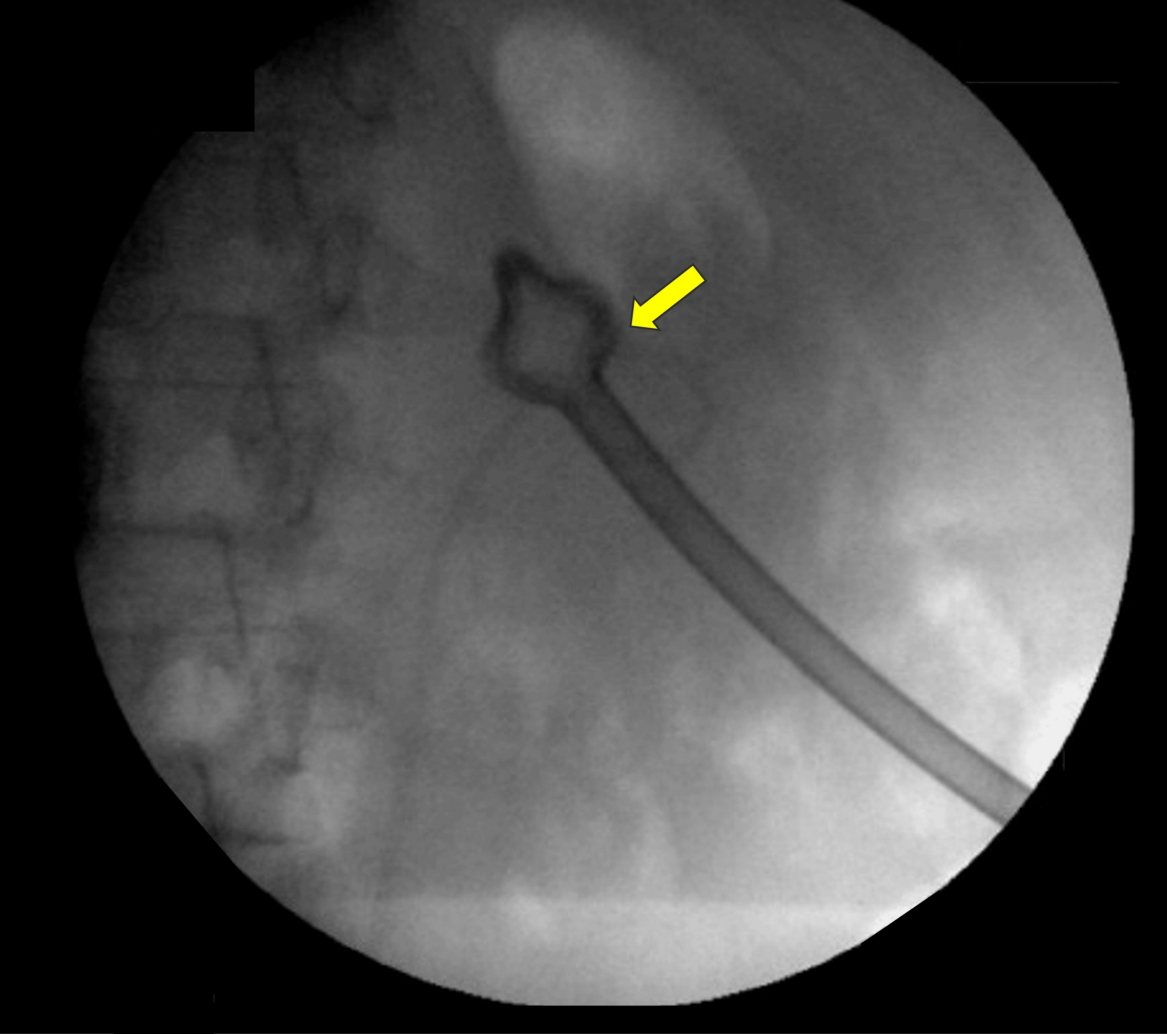
Intraoperative X-ray at the end of the PNL procedure showing placement of the Malecot catheter and the JJ stent (arrow). PNL, percutaneous nephrolithotomy; JJ, double-J

A 47-year-old man initially presented with a greater than 2 cm left renal pelvis stone. In January 2023, a JJ stent was inserted to relieve obstruction of an infected left kidney. The stent was exchanged in November 2023 due to heavy encrustation of the upper coil in preparation for a planned PNL. However, surgery was delayed due to social circumstances. In April 2024, the patient underwent left-sided PNL via a lower calyceal puncture. At the end of the procedure, an antegrade JJ stent and a Malecot catheter were placed, and plans were made to remove the catheter the following day (Figure 1).

**Figure 2:**
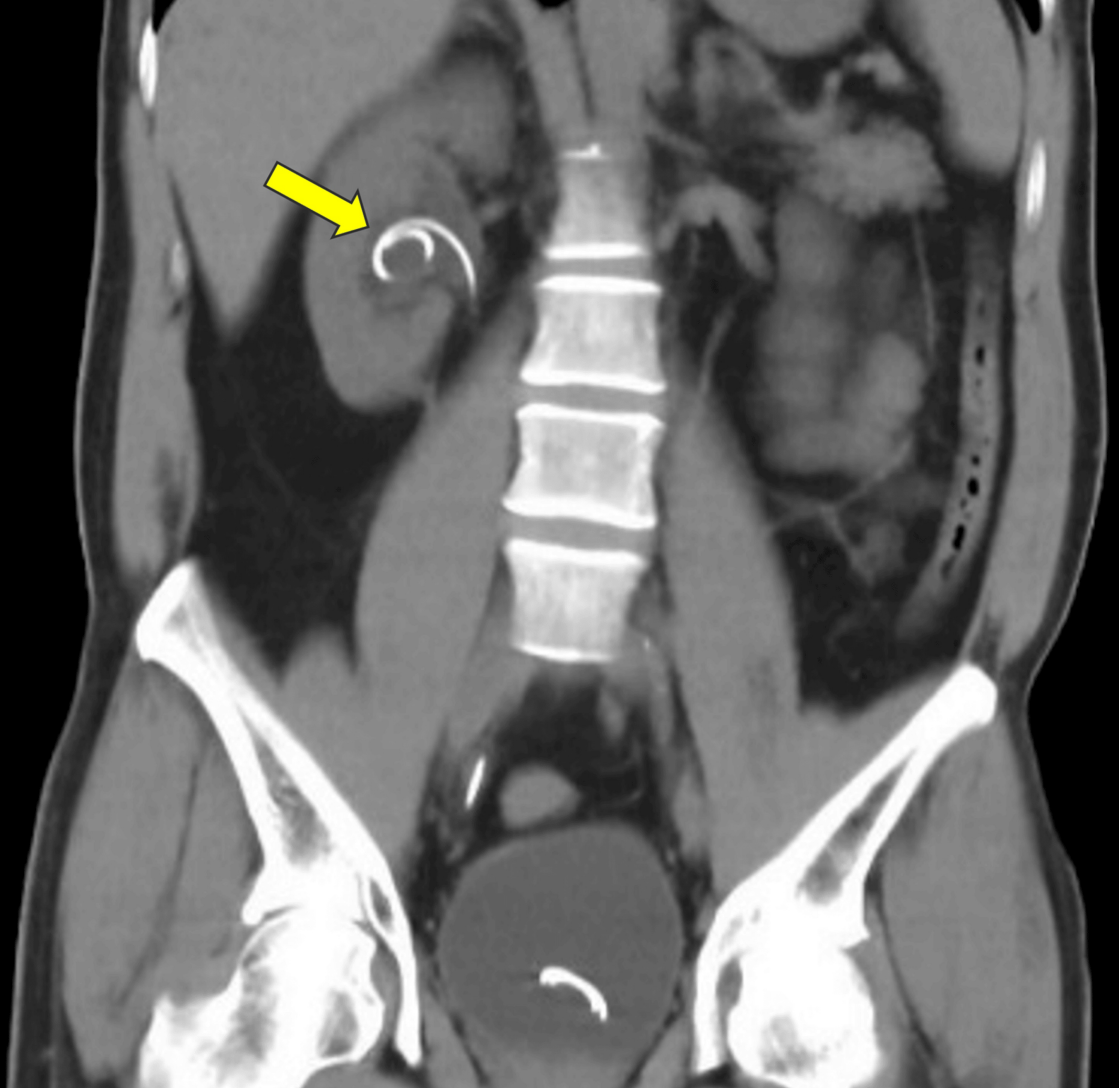
A follow-up scan (on May 9, 2024) showing the JJ stent in place (arrow). The upper coil is correctly positioned within the renal pelvis without evidence of encrustation. JJ, double-J

A follow-up scan showed that the JJ stent remained in place, with the upper coil correctly positioned in the renal pelvis and no encrustation evident (Figure 2).

**Figure 3:**
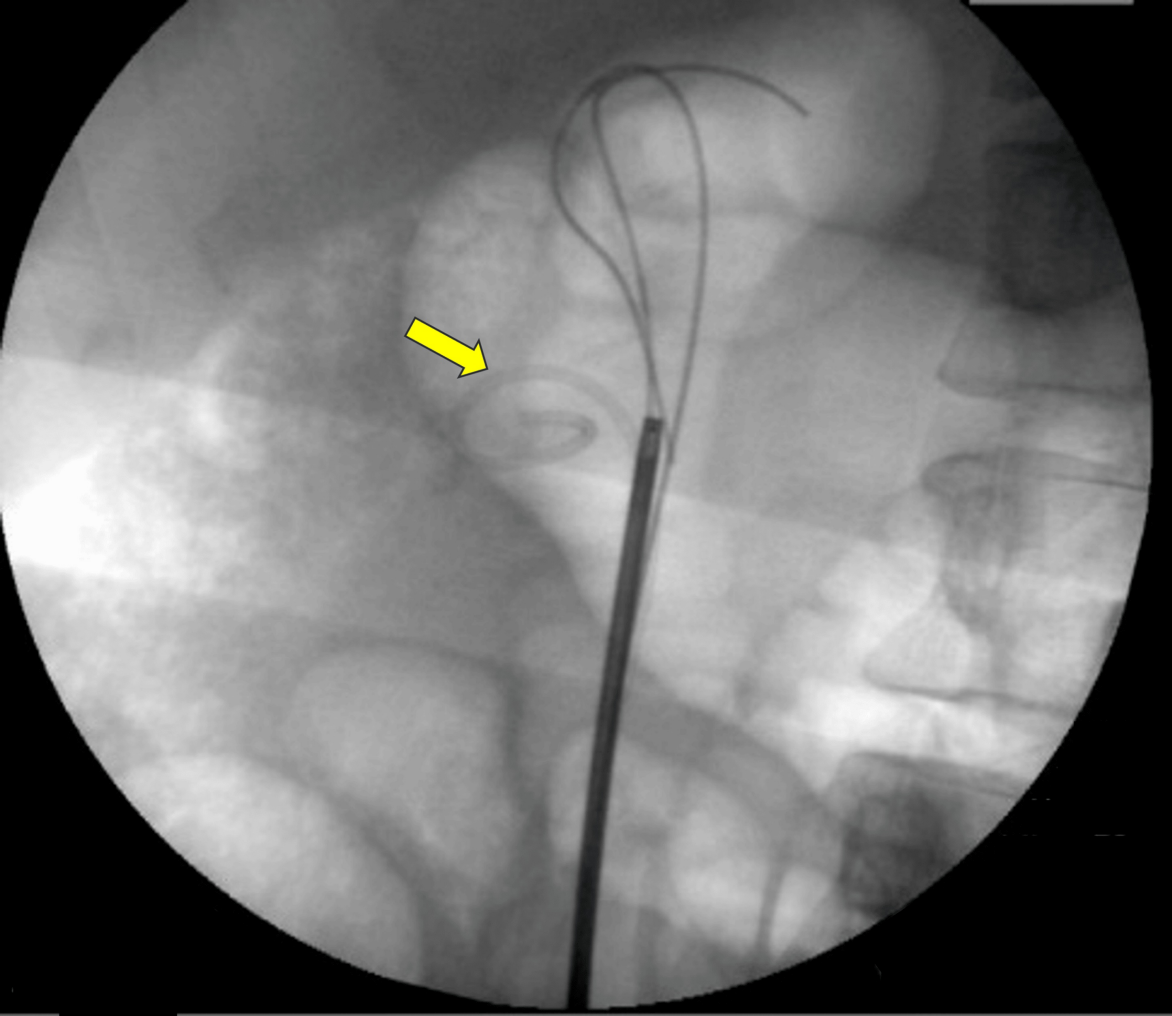
Intraoperative X-ray showing an attempt to gain access to the renal pelvis during failed stent removal (arrow).

A residual proximal ureteric stone was noted, requiring further intervention. In September 2024, a failed attempt was made to remove the JJ stent after securing a guidewire alongside it. A semirigid ureteroscope (URS) was used to extract small stone fragments surrounding the stent, but the stent itself remained in place (Figure 3).

**Figure 4:**
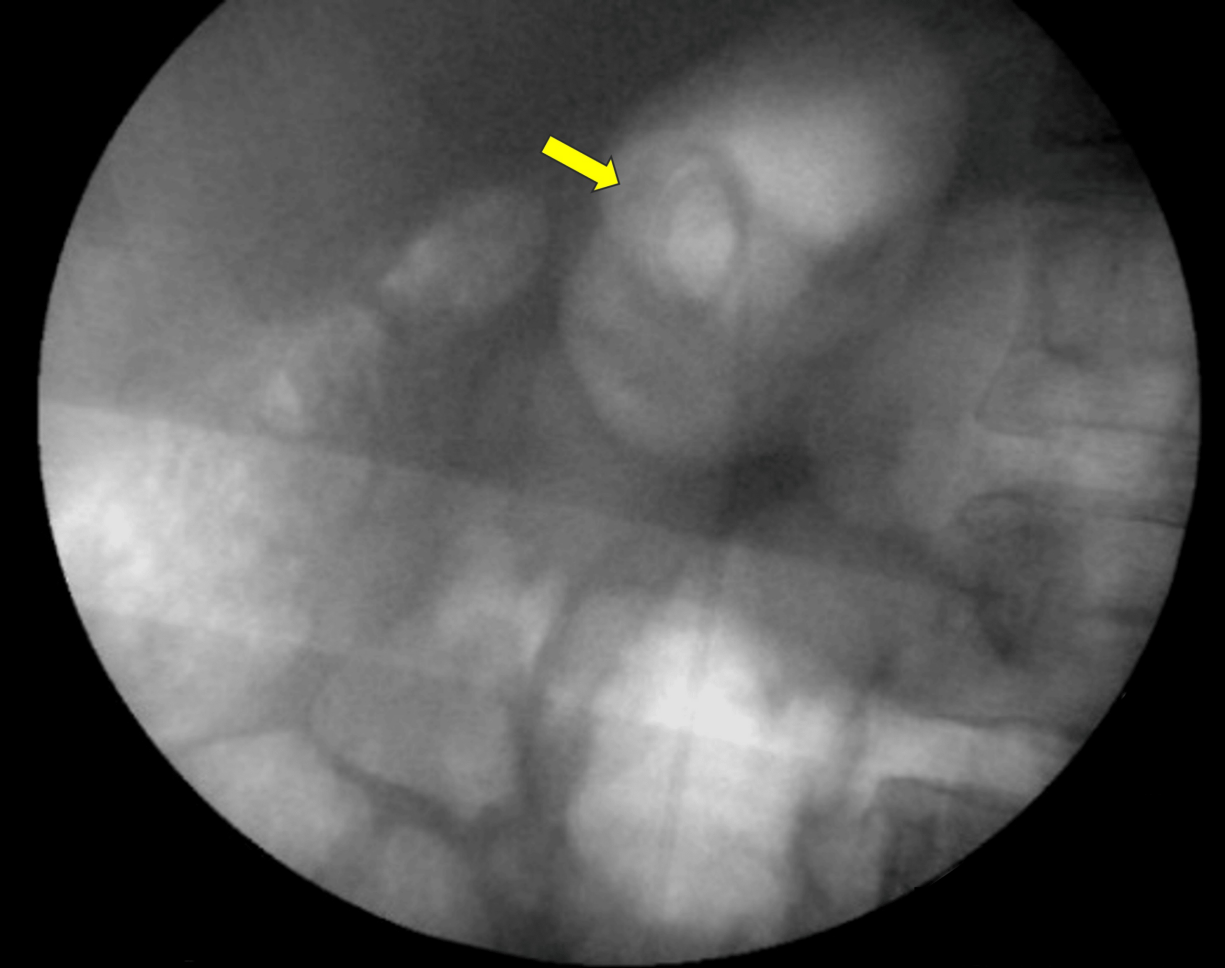
Intraoperative X-ray confirming the complete removal of all components of the retained JJ stent (arrow). JJ, double-J

Flexible URS revealed that the upper coil was embedded in the renal parenchyma, likely at the site of the prior puncture. Holmium laser, in dissection settings 0.5J and 8Hz, was employed to release the embedded stent from the renal parenchyma. All stent fragments were successfully removed and replaced with a new stent (Figure 4). Patient's postoperative outcome and follow-up were unremarkable.

## Discussion

JJ stents or ureteric catheters may serve as alternative drainage methods following tubeless PNL, providing benefits such as reduced postoperative discomfort, decreased analgesic use, and shorter hospital stays [12]. In this case, a Malecot catheter was used with the JJ stent to ensure adequate drainage of the pelvicalyceal system.

Few reports in the literature describe embedding of the JJ stent’s upper coil within the renal parenchyma at the site of renal pelvis perforation, likely due to fibrous tissue formation during healing. One such case required laser dissection to free the stent, while another necessitated a repeat PNL procedure [13]. In contrast, our case did not involve a reported perforation of the renal pelvis during the initial surgery. The embedded upper coil was localized to the anatomical site of the puncture, suggesting that overlapping of the JJ stent and Malecot catheter may have contributed. Partial displacement of the JJ stent during blind removal of the Malecot catheter, performed without fluoroscopic guidance, may have led to its misplacement.

Complications such as encrustation, migration, fracture, stone formation, penetration into adjacent organs, urinary tract infections, ureteral erosion, and fistula formation are more frequently observed when stents remain in place for over seven months [14-16]. However, in this case, the described complication occurred within five months of stent placement.

## Conclusions

When using a JJ stent combined with a Malecot catheter or pigtail drain, the latter should always be removed under fluoroscopic guidance to prevent inadvertent displacement. Repositioning the antegrade JJ stent after placement may help ensure that the upper coil is not located near the site of prior puncture or injury. These precautions may reduce the risk of rare but serious complications, such as parenchymal embedding of the stent. Using URS alongside the retained stent is a feasible option for diagnosis of the cause. Laser could be used as a minimal invasive tool for the management of the cause, for stent encrustation or stones embedded within the renal parenchyma, rather than open exploration that might carry major complications. However, the application of laser to cut through the renal parenchyma is rarely used and still requires more research to evaluate its safety and efficacy.
